# Addition of Bevacizumab to Vinorelbine-Platinum combination is efficacious in Heavily Pretreated HER2-Negative Metastatic Breast Cancer

**DOI:** 10.7150/jca.105199

**Published:** 2025-02-11

**Authors:** I-Wei Ho, Yi-Ru Tseng, Chun-Yu Liu, Yi-Fang Tsai, Chi-Cheng Huang, Ling-Ming Tseng, Ta-Chung Chao, Jiun-I Lai

**Affiliations:** 1 Division of Medical Oncology, Department of Oncology, Taipei Veterans General Hospital, Taipei, Taiwan.; 2 Institute of Clinical Medicine, National Yang-Ming Ming Chiao Tung University, Taipei, Taiwan.; 3 Comprehensive Breast Health Center, Taipei Veterans General Hospital, Taipei, Taiwan.; 4 School of Medicine, College of Medicine, National Yang-Ming Chiao Tung University, Taipei, Taiwan.; 5 Division of Breast Surgery, Department of Surgery, Taipei Veterans General Hospital, Taipei, Taiwan.; 6 Division of Cancer Prevention, Department of Oncology, Taipei Veterans General Hospital, Taipei, Taiwan.

**Keywords:** platinum, vinorelbine, metastatic breast cancer, bevacizumab, MAPK

## Abstract

**Introduction:** Despite rapidly improving therapeutics, challenges remain in the treatment of advanced breast cancer. Vinorelbine, a semisynthetic vinca alkaloid, is effective and well-tolerated in breast cancer treatment. The combination of vinorelbine and platinum-combination is a well-tolerated but underreported chemotherapy regimen. Bevacizumab, a VEGF-neutralizing antibody, has shown efficacy in HER2-negative metastatic breast cancer (mBC) when combined with chemotherapy. In this study we aimed to investigate the clinical and molecular effects of vinorelbine-platinum in heavily pretreated HER2-negative mBC, as well as the impact of adding bevacizumab.

**Material and methods:** We conducted a retrospective study at Taipei Veterans General Hospital to evaluate the effectiveness of the vinorelbine-platinum regimen in heavily pretreated HER2-negative mBC patients from 2016 to 2020, with a portion of patients receiving additional bevacizumab. To model the molecular perturbations at a cellular level, transcriptional profiling of a triple negative breast cancer cell line treated with cisplatin-vinorelbine was done by RNA-sequencing.

**Results:** The cohort included 54 patients. 50% of the patients received ≥ 5 lines of systemic treatment in the metastatic setting. All the patients had received anthracyclines and taxane. In patients treated with vinorelbine-platinum combination, the median progression-free survival (PFS) and overall survival (OS) were 2.3 and 7.3 months, respectively. With bevacizumab, median PFS improved to 4.1 months. Objective response rate (ORR) and disease control rate (DCR) without bevacizumab were 11.1% and 27.7%, respectively, improving to 25% and 83.3% with bevacizumab. Adverse events occurred in 37.0% of patients, with no grade IV events reported. Transcriptional profiling revealed significant downregulation of MAPK pathway, angiogenesis, and growth factor signaling related genes.

**Conclusion:** The vinorelbine-platinum regimen, particularly with bevacizumab, shows potential efficacy even in heavily pretreated HER2-negative metastatic breast cancer patients. Molecular analyses of treated cells highlight potential targets and mechanisms of action, providing a basis for future therapeutic strategies.

## Introduction

Breast cancer is one of the most common cancers worldwide, with over 2.3 million new diagnoses and 665,000 deaths reported globally in 2022^1^. Significant advances over the past two decades have dramatically improved outcomes across the three main subtypes: hormone receptor-positive (HR+), HER2-positive, and triple-negative breast cancer (TNBC). In metastatic breast cancer (mBC), overall survival (OS) now extends to 4-5 years for HR+ patients, largely due to the introduction of CDK4/6 inhibitors 2, and dual HER2 blockade with pertuzumab/trastuzumab has significantly benefited those with HER2-positive disease 3. For metastatic TNBC, the combination of immunotherapy and chemotherapy has improved survival to approximately 2 years in patients with high PD-L1 expression 4 Despite these advances, the five-year survival rate for mBC remains at approximately 30% 5. However, treatment resistance eventually limits options, particularly in heavily pretreated patients who have exhausted standard lines of therapy.

Vinorelbine, a synthetic vinca alkaloid chemotherapeutic agent, inhibits microtubule formation during mitosis, disrupting cell division.^6^ Its demonstrated efficacy, favorable tolerability, and relatively low toxicity make vinorelbine a common choice for treating advanced metastatic breast cancer, particularly in fragile patients or palliative care settings 7, 8 Additionally, vinorelbine plays a key role in metronomic therapy for breast cancer^9^, and has shown superior efficacy compared to weekly paclitaxel in a phase 2 trial.^10^.

Platinum agents, such as cisplatin and carboplatin, have an established role in breast cancer treatment, particularly for TNBC. These drugs are now considered standard of care in the neoadjuvant treatment of non-metastatic TNBC and for metastatic TNBC in patients with BRCA1/2 mutations 11-13. While the combination of vinorelbine and platinum is commonly used in lung cancer^14^, its application in breast cancer has been relatively underreported over the past two decades 15, 16. This gap led us to investigate whether the combination of vinorelbine and platinum could offer a viable treatment option for heavily pretreated breast cancer patients while maintaining an acceptable toxicity profile.

Angiogenesis is a key factor in the progression of mBC. 17 Elevated serum levels of vascular endothelial growth factor (VEGF) in mBC patients have been linked to poorer clinical outcomes.^18^ Bevacizumab, a VEGF-neutralizing antibody, plays a crucial role in inhibiting tumor angiogenesis, thereby slowing tumor progression.^19^ Previous meta-analyses have demonstrated the efficacy of bevacizumab in HER2-negative mBC when combined with chemotherapy, leading to improved progression-free survival (PFS) and objective response rates (ORR) 20. In first-line treatment of HER2-negative mBC, the addition of bevacizumab to chemotherapy resulted in a PFS of 9.2 months and a response rate of 49% 21, with particular benefit observed in high-risk groups, including patients with metastatic TNBC.

Thus, this study seeks to explore whether the combination of vinorelbine and platinum could offer a viable therapeutic alternative for heavily pretreated patients with mBC. Additionally, we aim to investigate whether the addition of bevacizumab to this regimen may further enhance its efficacy by targeting angiogenesis. By examining these combinations, our research will contribute to understanding the potential benefits and limitations of these treatment strategies in mBC patients with limited options.

## Material and methods

### Study design and setting

This retrospective cohort study was conducted at Taipei Veterans General Hospital, Taiwan, to evaluate the efficacy of salvage chemotherapy regimens involving the vinorelbine-platinum combination. Additionally, the study aimed to compare treatment responses, PFS, OS, prognostic factors, and potential adverse outcomes. The focus was on patients with HER2-negative metastatic breast cancer who received salvage chemotherapy between March 2016 and December 2020. The study also investigated the impact of adding bevacizumab to the vinorelbine-platinum regimen. Data were extracted from the hospital's electronic medical records, encompassing a wide range of clinical information, including vital signs, symptoms, diagnostic procedures, workup details, and patient management.

Patients included in the study had confirmed diagnoses via histopathology, and those who were referred to other hospitals for chemotherapy were excluded. HER2 negativity was defined as an immunohistochemistry (IHC) score of 1+ or 0. For cases with an IHC score of 2+, HER2 negativity was confirmed with a negative fluorescence in situ hybridization (FISH) result. Additional data included age at mBC diagnosis, sites of metastasis, prior chemotherapy regimens, treatment sequences, and adverse events. Institutional Review Board (IRB) approval was obtained from Taipei Veterans General Hospital (IRB TPEVGH No. 2023-09-007BC).

Key outcome measures included ORR, disease control rate (DCR), PFS, and OS, all assessed according to Response Evaluation Criteria in Solid Tumors (RECIST) version 1.1 22 Adverse events were also documented. OS was defined as the time from the initiation of vinorelbine-platinum chemotherapy to death from any cause, while PFS was measured from the start of treatment to disease progression or death.

### Statistical analysis

All statistical analyses were conducted using SPSS version 26. A two-sided p-value of less than 0.05 was considered statistically significant. Survival curves were generated using the Kaplan-Meier method and compared using the log-rank test. Univariate and multivariate analyses were performed to evaluate the associations between study attributes and PFS and OS. The Cox proportional hazards model was applied for both univariate and multivariate assessments of prognostic factors related to PFS and OS. The results were expressed as hazard ratios (HRs) with corresponding 95% confidence intervals (CIs) to quantify the risk of survival decline.

### Cell culture and drug treatment

The HR (+) breast cancer cell lines MDA-MB-231 (purchased from ATCC Cell Lines (https://www.atcc.org/) were maintained in Dulbecco's Modified Eagle Medium (DMEM, Gibco) respectively. All culture medium contained 10% Fetal Bovine Serum (FBS, Gibco) and 1% Penicillin Streptomycin (P/S, Gibco). Cells were incubated at 37°C, 5% CO2 under standard molecular biology conditions. Vinorelbine and cisplatin were purchased from Sigma-Aldrich (Missouri, United States) and diluted, stored per manufacturer instructions until use in cell culture.

### RNA-sequencing

MDA-MB-231 cells (5 to 7 × 10⁵ per well) were seeded onto 6 cm dishes and treated with either 7 μM cisplatin + 14 μM vinorelbine or vehicle control (DMSO) for 24 hours. After treatment, cells were washed with PBS, and RNA was extracted using the Total RNA Isolation Kit (NovelGene). The quantity of RNA was measured with a NanoDrop spectrophotometer (Thermo Fisher Scientific), and samples with an A260/A280 ratio over 1.9 were used for further analysis. The RNA was stored at -80°C until library preparation.

Libraries were prepared using the TruSeq Stranded mRNA Sample Preparation Kit (Illumina, San Diego, CA) from 500 ng of purified total RNA, following the manufacturer's protocol with a reduced reaction volume. The quality of the finished cDNA libraries was assessed using a Bioanalyzer, and library concentration was quantified using the Quant-iT dsDNA Assay Kit (Thermo Fisher Scientific, Waltham, MA). Uniquely indexed libraries were pooled based on the quantification results and further quantified by qPCR using the Kapa Biosystems Library Quantification Kit (Wilmington, MA) at the Molecular Biology Core Genomics Facility, Dana-Farber Cancer Institute. The pooled samples were then sequenced on an Illumina NovaSeq 6000 system with paired-end reads.

### Data Processing and Differential Expression Analysis

The sequencing reads were processed using the bcbio-Nextgen toolkit (version 1.0.3a, https://github.com/chapmanb/bcbio-nextgen) with the following steps: (1) Reads were trimmed and clipped for quality control using cutadapt v1.12; (2) Read quality was assessed for each sample using FastQC 0.11.5; (3) High-quality reads were aligned to the human genome assembly GRCh37 using STAR 2.5.3a, generating BAM files; (4) BAM files were processed using DEXSeq-COUNT 1.14.2 to calculate raw counts, transcripts per million (TPM), and reads per kilobase of transcript per million mapped reads (RPKM). For differential expression analysis, the edgeR package (version 3.18.1, R version 3.2.1) was used to calculate log fold changes, p-values, and false discovery rates (FDR) (McCarthy *et al.*, 2012; Robinson *et al.*, 2010). The RNA-seq data were deposited into the Gene Expression Omnibus (GEO) database under the accession number GSE267621.

### Pathway analysis and data processing

Heatmaps and gene ontology (GO) enrichment analyses were generated using the R packages “ClusterProfiler,” “enrichplot,” and “ComplexHeatmap.” For additional functional annotation and pathway analysis, DAVID analysis was performed using the online DAVID tool (https://david.ncifcrf.gov/tools.jsp) 23). Differentially expressed genes (DEGs) were identified using the DESeq2 package with the following criteria: a combined sum of counts greater than 20 across 2 DMSO control samples and 2 drug-treated samples, and an adjusted p-value (p-adj) of less than 0.05.

## Results

### Clinical characteristics of a HER2 negative metastatic breast cancer cohort

A total of 54 patients diagnosed with HER2 negative mBC and treated with the vinorelbine/platinum regimen were included in the study. The baseline characteristics of these patients are detailed in **Table 1**. The entire cohort comprised of female participants. The average age was 57.2 ± 8.7 years. Among them, 35 patients (64.8%) exhibited ER and/or PR positivity, while 19 patients (35.2%) were categorized as TNBC. More than 60% of patients had either bone, lung, or liver metastases, reflecting a cohort with heavy disease burden. All patients had received prior treatment with anthracyclines and taxane. 50% of all patients had previously received more than five lines of chemotherapy. 20 patients (37%) had *de novo* metastatic disease upon diagnosis, while 63% had recurrent disease. None of the patients in our cohort had received platinum-based therapy previously, including in the neoadjuvant setting. Other clinical details are described in **Table 1**.

### Clinical efficacy and adverse effects of the platinum plus vinorelbine, with or without addition of bevacizumab

**Table 2** presents the clinical efficacy data. The median PFS was recorded at 2.3 months, while the median OS was 7.3 months. With the addition of bevacizumab, median PFS was extended to 4.1 months (HR: 0.54, p =0.04), and OS increased to 12.4 months. Among the patients, 11.1% (6/54) achieved a partial response (PR), 16.7% (9/54) exhibited stable disease (SD), and 72.2% (39/54) demonstrated progressive disease (PD). The calculated rates for ORR and DCR were 11.1% and 27.7%, respectively; and were increased to 25% and DCR to 83.3%, respectively. Interestingly, TNBC patients had a longer median OS of 12 months, while HR+ patients had an OS of 6.6 months.

**Figure 1:**
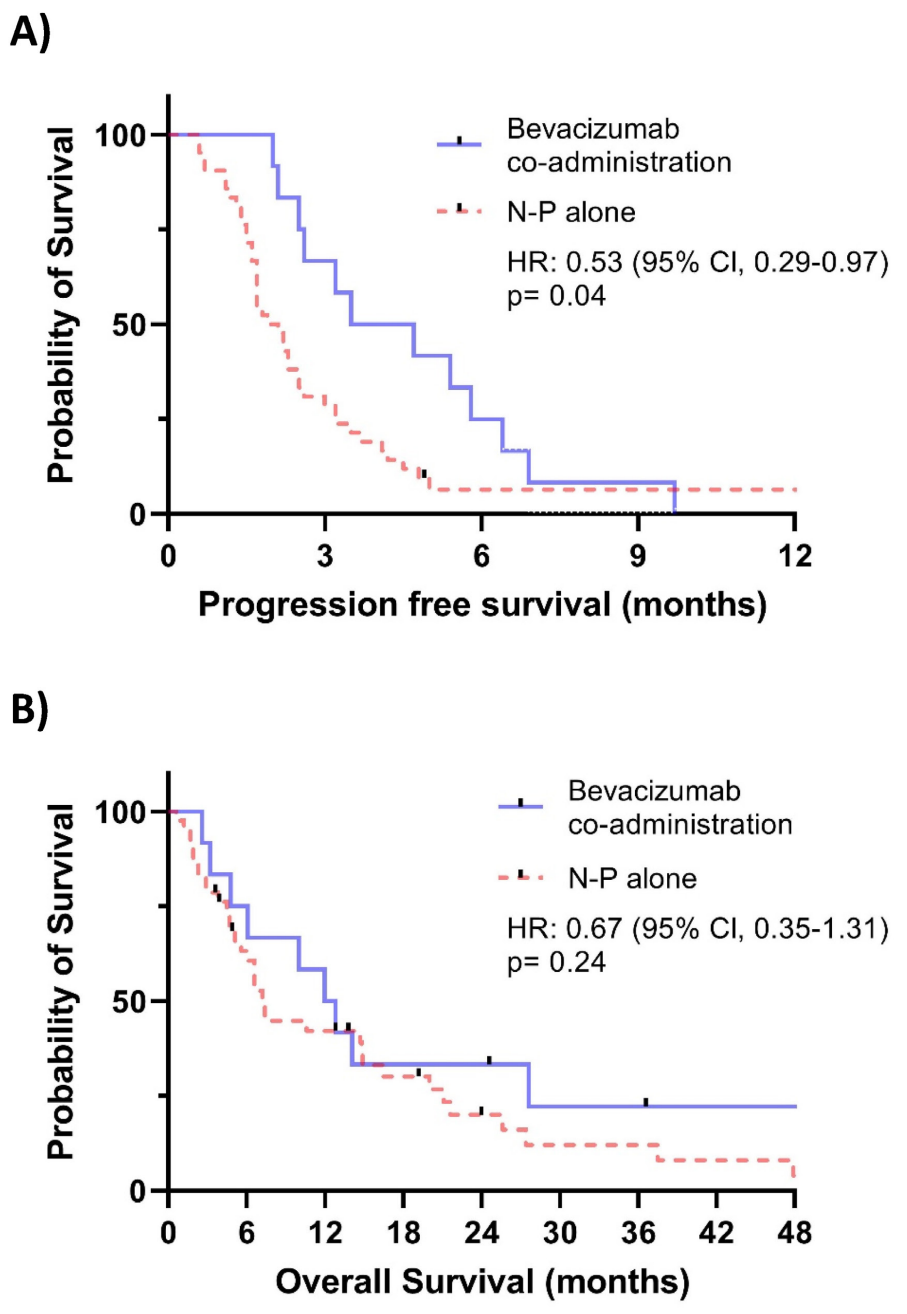
A. Kaplan-Meier plot comparing progression-free survival between patients receiving Bevacizumab co-administration and those receiving NP alone. B. Kaplan-Meier plot comparing overall survival between patients receiving Bevacizumab co-administration and those receiving NP alone.

Of the 54 patients included in the study, 12 (22.2%) received additional bevacizumab treatment. Kaplan-Meier plots with log-rank tests are shown in **Figure 1**. **Figure 1A** demonstrated a significant benefit in PFS associated with the addition of bevacizumab (HR = 0.53, 95% CI: 0.29-0.97, p = 0.04). However, no significant improvement in OS was observed in **Figure 1B**.

**Figure 2:**
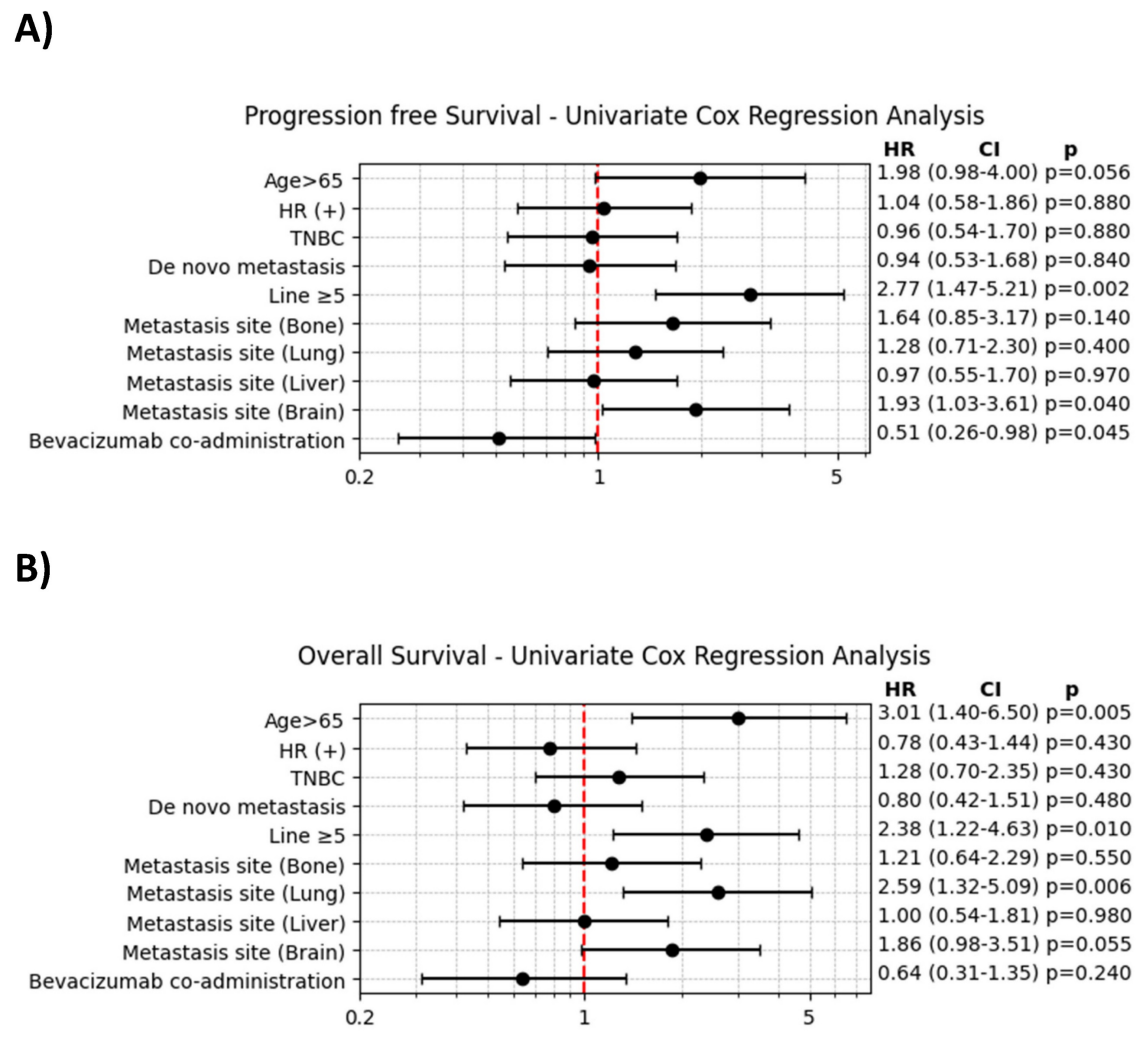
A. Univariate Cox regression analysis of progression-free survival. B. Univariate Cox regression analysis of overall survival.

The results of the univariate Cox regression analysis are shown in **Figure 2**. For PFS, significant factors associated with an increased HR included age > 65 and the presence of brain metastases. In contrast, the addition of bevacizumab significantly decreased the HR for progression (HR = 0.51, 95% CI: 0.26-0.98, p = 0.045). For OS, age > 65, the presence of lung metastasis, and prior treatment with ≥ 5 lines of chemotherapy were significantly associated with an increased HR for mortality.

**Figure 3:**
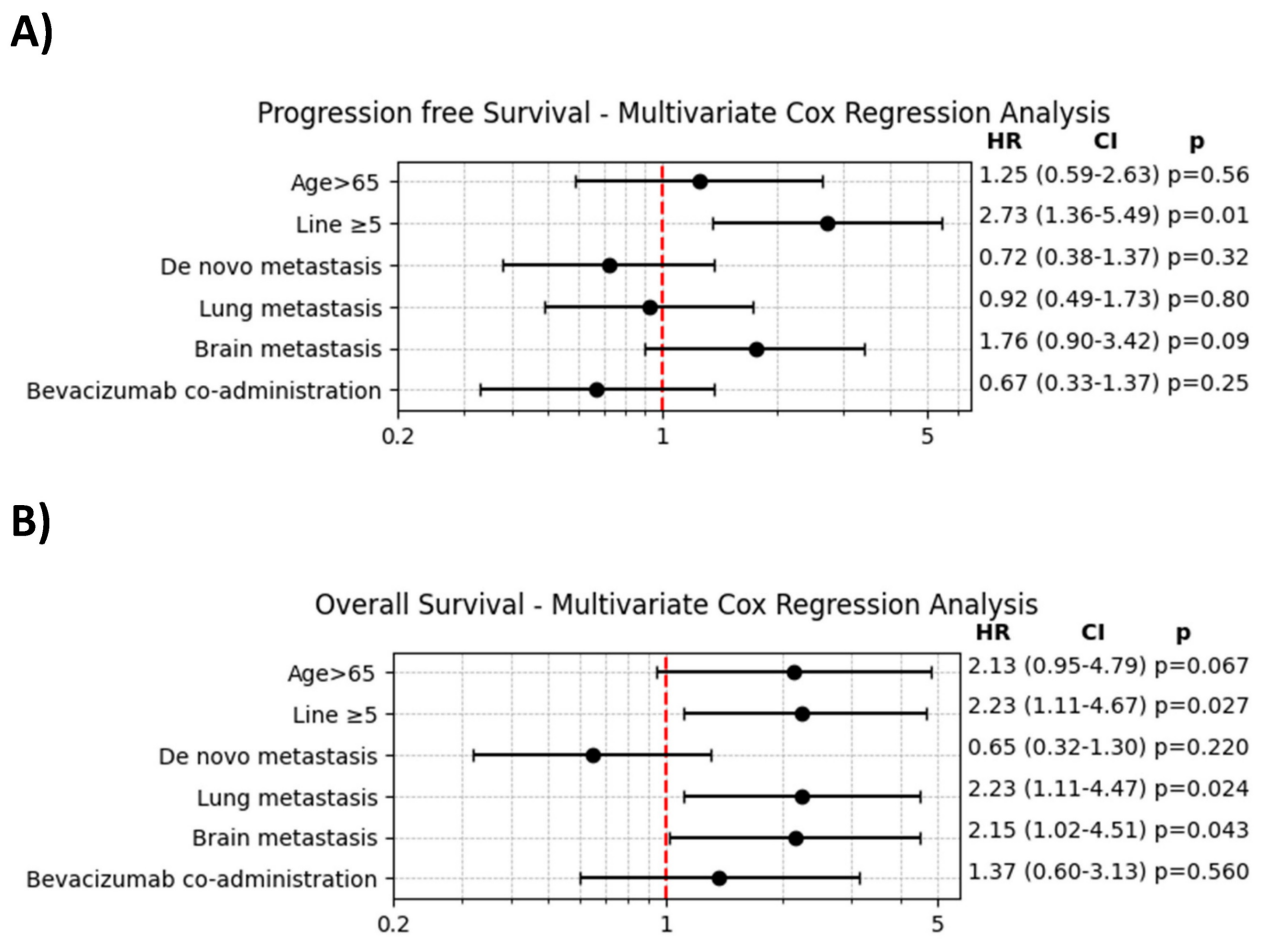
A. Multivariate Cox regression analysis of progression-free survival. B. Multivariate Cox regression analysis of overall survival.

The findings from the multivariate Cox regression analysis, presented in **Figure 3**, indicated that prior treatment with ≥ 5 lines of chemotherapy was significantly associated with both decreased PFS and OS. However, the addition of bevacizumab did not show a significant impact on either PFS or OS in the multivariate analysis.

Within our cohort of 54 patients, ≥ grade 3 adverse events were documented in 20 individuals (37.0%), described in **Table 3**. Most of the adverse effects were related to bone marrow toxicity, including grade III neutropenia accounting for 25.9% of cases, and grade III anemia in 12.5%. Importantly, no fetal adverse events were reported in the study.

### Transcriptional profiling of TNBC cells treated with cisplatin-vinorelbine reveals molecular insights of cytotoxic mechanisms

**Figure 4:**
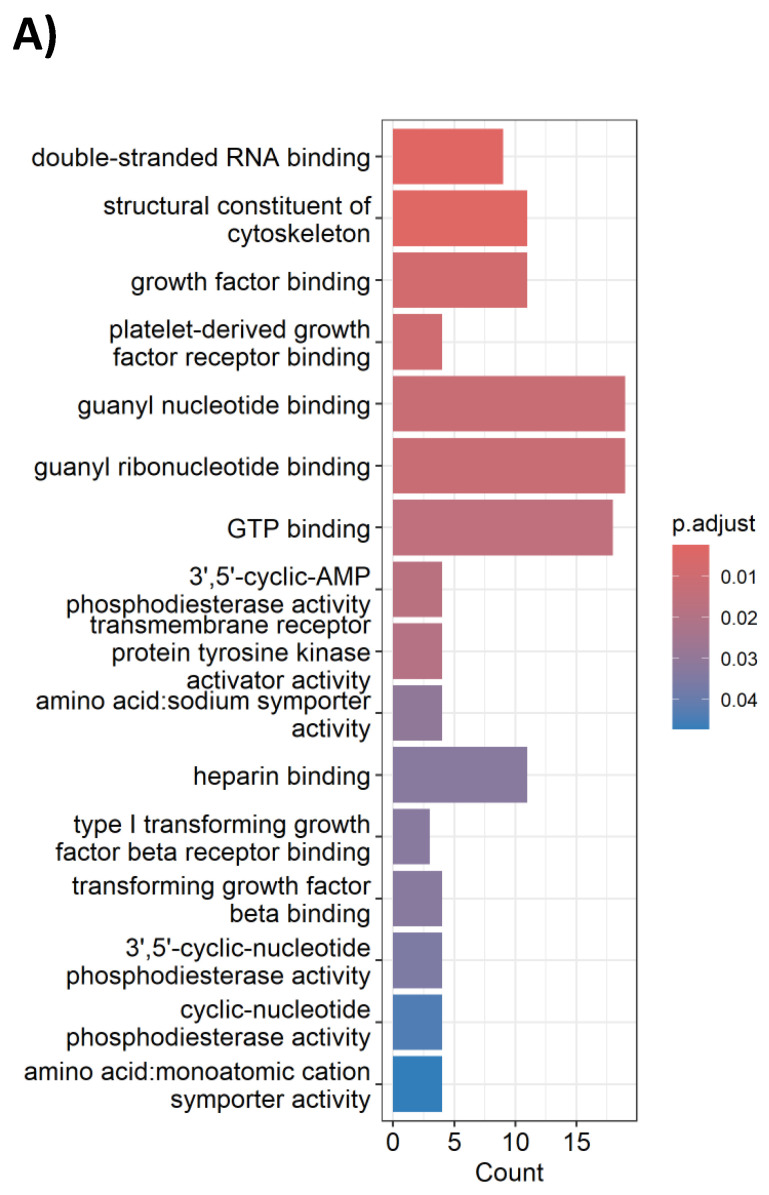

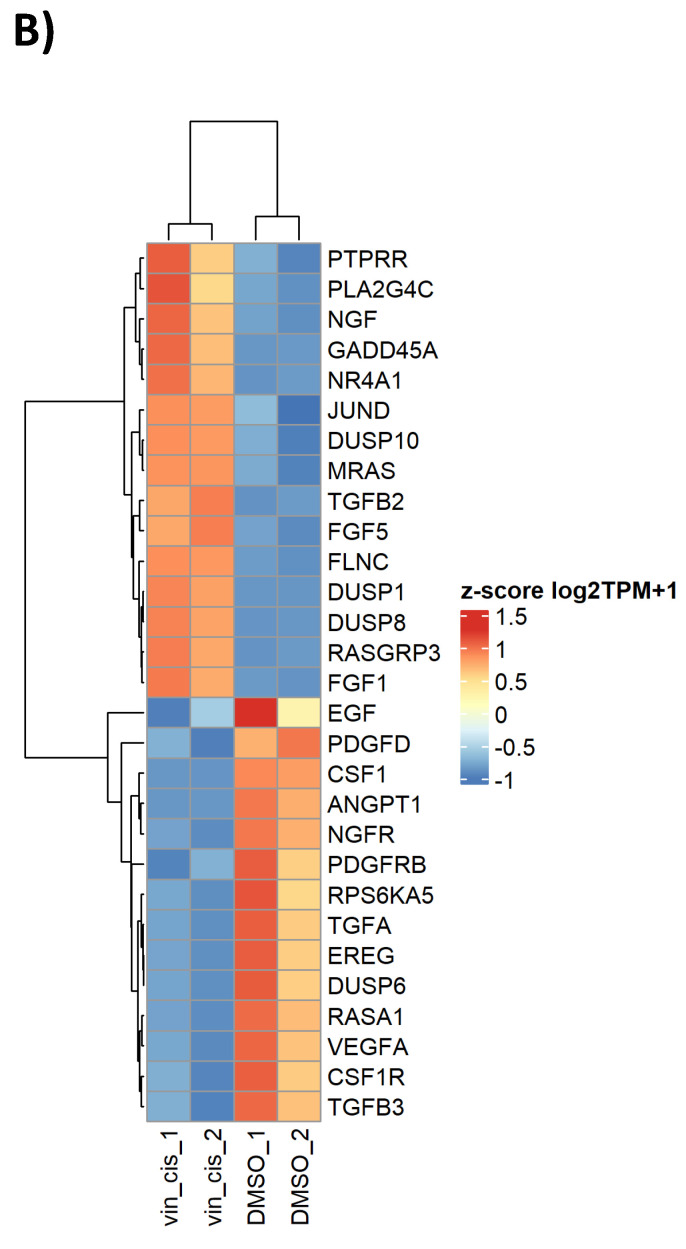
A. pathway analysis by DAVID, the top enriched pathways were the MAPK pathway and the Ras signaling pathway. B. Genes from the MAPK pathway, including EGF, CSF1, NGFR1, TGFA, VEGFA, and CSF1R, were downregulated by the cisplatin-vinorelbine treatment.

To model the molecular perturbations by cisplatin-vinorelbine combination, we treated the TNBC MDA-MB-231 cell line with a combination of cisplatin plus vinorelbine, in comparison to vehicle treatment. Total RNA extracted from treated cells underwent RNA-sequencing for transcriptional profiling. Differential expression genes (DEGs) were analyzed for enriched pathways. We observed that in gene ontology (GO) analysis, cisplatin-vinorelbine combination resulted in strong impact of growth factor binding, PDGFR binding, TGF-beta binding, as well as other pathways (**Table 4**). In pathway analysis by DAVID, the top enriched pathways were the MAPK pathway and the Ras signaling pathway (**Figure 4A**). Genes from the MAPK pathway, including EGF, CSF1, NGFR1, TGFA, VEGFA, and CSF1R, were downregulated by the cisplatin-vinorelbine treatment (**Figure 4B**), suggesting that platinum-vinorelbine regimen resulted in transcriptional suppression of many tumors related growth factor signaling pathways.

## Discussion

Despite advancements in therapies for mBC, treatment options for heavily pretreated patients remain limited, and clinical efficacy is often modest. A real-world study using the Flatiron database applied MONARCH 1 trial criteria to assess outcomes in heavily pretreated patients with HR (+) HER2(-) mBC receiving monotherapy with agents such as eribulin, vinorelbine, gemcitabine, or capecitabine. In the cohort, the median OS was 13.6 months 24. Similarly, a separate retrospective study of mBC patients treated with eribulin reported a median OS of 7 months and a PFS of 3 months 25. These results align with our findings, where our cohort, consisting of heavily pretreated patients, showed modest survival outcomes yet acceptable toxicity and tolerability.

To date, the optimal first-line regimen for metastatic TNBC consists of chemotherapy with or without immunotherapy or PARP inhibitors, depending on PD-L1 expression and germline BRCA mutation status. Beyond first-line therapy, sacituzumab govitecan has demonstrated benefit in later lines of treatment. 26, 27 However, there is no clear consensus on subsequent chemotherapy regimens, and metastatic TNBC remains incurable, highlighting the urgent need for novel therapies. Eribulin, capecitabine, ixabepilone, and platinum-based therapies are all considered reasonable options, yet no single regimen is regarded as superior in this setting 26.

In our study, we observed that the addition of bevacizumab appeared to provide a therapeutic advantage, as indicated by improved PFS in univariate analysis. However, this effect was not retained in multivariate analysis, likely due to the influence of confounding factors such as age, metastasis sites, and prior lines of therapy. The small sample size may have further limited the power of the multivariate analysis to detect a significant independent effect of bevacizumab. Despite this, our findings align with previous studies, such as the ATHENA study, in which the TNBC subgroup receiving first-line bevacizumab plus chemotherapy had a PFS of 7.2 months and an OS of 18.3 months, with an ORR of 49% 28. Moreover, our RNA-seq data suggests that bevacizumab's efficacy may be linked to its modulation of angiogenesis, offering a potential strategy to enhance current treatment approaches in TNBC.

The vinorelbine-platinum combination has demonstrated efficacy in TNBC in prior studies, even in heavily pretreated patients 29, 30. Our findings extend these observations by showing a comparable response in both TNBC and HR-positive patients, with an ORR of 11.1% and a DCR of 27.7%. Notably, the co-administration with bevacizumab enhanced these results, increasing the ORR to 25% and the DCR to 83.3%. Despite a lower response rate compared to prior studies 29, 30, it is critical to acknowledge that half of our cohort had undergone more than five lines of prior therapy. This heavily pretreated status makes the observed improvements with bevacizumab particularly notable, as it significantly improved disease control in this frail patient population.

The MAPK signaling pathway plays a crucial role in breast cancer development, particularly influencing the expression of estrogen receptor (ER), progesterone receptor (PR), and HER2. It is closely associated with the invasion, metastasis, and prognosis of TNBC.^31^ Elevated MAPK activity correlates with shorter survival times in TNBC patients, suggesting its potential as a prognostic indicator.^31^, ^32^ Various mitogens such as TGF-α, EGF, VEGF, and PDGF-β bind to their respective receptors, triggering RAS activation, which subsequently stimulates the MAPK pathway.^33^ In our RNA sequencing analysis of TNBC cell lines treated with a cisplatin-vinorelbine combination, we observed downregulation of the MAPK pathway. Notably, targeting various steps in the MAPK pathway with BRAF plus MEK inhibitors has been established as the standard of care for patients with advanced-stage melanoma harboring BRAF V600 mutations.^34^ MEK inhibitor Trametinib has been proposed to possess activity towards TNBC in phase 2 study.^35^ We hypothesize that combining this approach with bevacizumab could yield synergistic antitumor effects through vertical inhibition. Interestingly, VEGF-A expression was downregulated in our RNA-seq data. There have been conflicting reports on whether high VEGF-A in cancer shows more sensitivity 36 or more refractoriness 37. A prospective study using a combination of paclitaxel and bevacizumab in breast cancer discovered that VEGF-A expression was not correlated with treatment outcome, however the study revealed potential biological and prognostic roles of VEGF-VEGFR combinations 38. In summary, our RNA-seq study reveals downregulation in multiple RTK gene expression, indicating that the biological subsequence of the platinum/vinorelbine combination may extend beyond the canonical role of apoptosis induction in cancer cells. This provides much insight for further research.

Given that vinorelbine targets microtubules, other anti-microtubule agents such as eribulin, ixabepilone, and taxane also play significant roles, not only in first-line but even in heavily pretreated patients 26, 39, 40. It is intriguing to speculate that newer agents, such as antibody-drug conjugates with tubule-targeting payloads like enfortumab vedotin, may demonstrate clinical activity in metastatic TNBC, with or without the addition of bevacizumab 41. Another important drawback is that our cell line used, MDA-MB-231, although a well-established TNBC cell line 42, may not be entirely reflective of the heavily pretreated and chemotherapy refractory status in our patient cohort. However, we maintain that our RNA-seq data still provides important molecular insight, especially the role of VEGF-A and RTK signaling offered by chemotherapy. The ideal follow up experiment would be to compare a cohort of newly diagnosed HER2-negative patients versus a heavily pretreated cohort to determine whether there are differences in RTK, especially angiogenesis signaling, to further validate the data in this study. This will be a much larger scale study that we hope to foresee in the future.

It is important to acknowledge that our study had a limited sample size, making it challenging to draw definitive conclusions regarding the specific characteristics of the vinorelbine-platinum regimen. Additionally, BRCA mutation testing was not conducted in our cohort, which could have provided further insights given the established sensitivity of BRCA-mutated breast cancers to platinum-based therapies. Furthermore, the transcriptional analysis relied on the MDA-MB-231 cell line, a triple-negative breast cancer model. While this cell line is a valuable tool for studying aggressive and treatment-resistant phenotypes, it does not fully capture the heterogeneity of HER2-negative breast cancer, particularly hormone receptor-positive subtypes. Future studies with larger, more diverse cohorts and the inclusion of multiple cell line models are warranted to better define the patient subgroups that may benefit most from vinorelbine-platinum therapy. Despite these limitations, we believe our study offers valuable insights into a cohort with an extremely poor prognosis and limited clinical options, contributing preliminary data that could inform the development of more effective systemic therapies.

## Conclusions

The vinorelbine/platinum regimen, particularly when combined with bevacizumab, demonstrates efficacy in heavily pretreated HER2-negative metastatic breast cancer patients. Adverse events associated with this treatment were manageable. Molecular analyses of treated cells have identified potential targets and mechanisms of action, laying the groundwork for the development of future therapeutic strategies.

## Figures and Tables

**Table 1:** Baseline characteristics

Characteristics	Total, N=54
**Age, mean ± SD**	57.2± 8.7
**Age Group**	
Age ≥ 65 yr, No. (%)	11 (20.4)
Age < 65, No. (%)	43 (79.6)
**Female, n (%)**	54(100)
**ER/PR status, n (%)**	
Triple negative	19 (35.2)
ER and/or PR positive	35 (64.8)
Hormone-Resistant status*	5 (14.3)
Prior CDK4/6 inhibitor	20 (57.1)
**Metastatic sites, n (%)**	
Bone	37(68.5)
Lung	35(64.8)
Liver	34(62.9)
Brain	15(27.7)
**No. of regimens for advanced disease, n (%)**	
2	3(5.6)
3	9(16.7)
4	15(27.7)
≥5	27(50)
**Combination with bevacizumab**	12(22.2)
***De novo* metastatic disease, No. (%)**	20(37.0)

Data are presented as mean ± SD for age and n (%) for others; SD: Standard deviation, Hormone-resistant status: Defined as progression within 6 months of a hormone-based regimen in patients with ER-positive and/or PR-positive group.

**Table 2:** Efficacy analysis

Endpoint	All patient N=54	Hormone positive N=35	Triple negative patients N=19	Bevacizumab co-administration N=12
PFS, months (median, range)	2.3 (0.6-40.1)	2.3 (0.6-12.3)	2.5 (0.6-40.0)	4.1 (2.0-9.7)
OS, months (median, range)	7.3 (0.6-61.0)	6.6 (1.2-61.0)	12.0 (0.9-47.9)	12.4 (2.6-61.0)
Best objective response, n (%)				
CR	0(0)	0(0)	0(0)	0(0)
PR	6(11.1)	4(11.4)	2(10.5)	3(25.0)
SD	9(16.7)	5(14.3)	4(21.1)	4(33.3)
PD	39(72.2)	26(74.3)	13(68.4)	5(41.7)
ORR	6(11.1)	4(11.4)	2(10.5)	3(25.0)
DCR	15(27.7)	9(25.7)	6(31.6)	7(58.3)

PFS, Progression-Free Survival; OS, Overall Survival; CR, Complete Response; PR, Partial Response; SD, Stable Disease; PD, Progressive Disease; ORR, Objective Response Rate (calculated as the sum of CR and PR); DCR, Disease Control Rate (calculated as the sum of CR, PR, and SD).

**Table 3:** Treatment emergent adverse events (TEAE)

TEAE	N (%)
Any adverse events ≥ 3	20 (37.0)
Grade 3	20 (37.0)
Grade 4	1 (1.9)
Grade 5	0 (0)
Hematological	
Neutropenia	14 (25.9)
Febrile neutropenia	1 (1.9)
Anemia	8 (14.8)
Thrombocytopenia	7 (13.0)
Renal and electrolyte imbalance	
Creatinine increased	1 (1.9)
Hypokalemia	5 (9.3)
Hypomagnesemia	1 (1.9)
Hyponatremia	2 (3.7)
Liver function disturbances	5 (9.2)
Body weight loss	5 (9.3)
Mucositis	3 (5.6)

**Table 4:** Gene ontology (GO) analysis

Term	Count	P Value	Fold Enrichment	FDR
MAPK signaling pathway	29	7.39E-06	2.568	6.80E-04
Ras signaling pathway	24	2.36E-05	2.71	1.30E-03
Rap1 signaling pathway	21	1.11E-04	2.665	4.70E-03
Lipid and atherosclerosis	18	0.003	2.231	4.10E-02
Legionellosis	8	0.005	3.807	5.70E-02
Alcoholic liver disease	13	0.007	2.44	7.00E-02

Cisplatin-vinorelbine combination resulted in strong impact of growth factor binding, PDGFR binding, TGF-beta binding, as well as other pathways
